# Fractal analysis as a useful predictor for determining osseointegration of dental implant? A retrospective study

**DOI:** 10.1186/s40729-021-00296-0

**Published:** 2021-02-25

**Authors:** Emrah Soylu, Aykağan Coşgunarslan, Selin Çelebi, Damla Soydan, Ahmet Emin Demirbaş, Osman Demir

**Affiliations:** 1 Faculty of Dentistry Department of Oral and Maxillofacial Surgery, Erciyes University, Kayseri, Turkey; 2 Faculty of Dentistry Department of Oral Maxillofacial Radiology, Erciyes University, Kayseri, Turkey; 3 Faculty of Medicine Departments of Bioistatistics, Tokat Gaziosmanpaşa University, Tokat, Turkey

**Keywords:** Osseointegration, Fractal analysis, Implant, Fractal dimension

## Abstract

**Purpose:**

The present study aimed at evaluating the effectiveness of fractal analysis on determining the osseointegration of dental implants.

**Material and methods:**

In a single center, retrospective clinical trial, patients with dental implants in the mandibular premolar/molar region, ASA I–II and < 65-year-old patients were included. Orthopantomograph (OPG) were taken before implant surgery (t0), within a week of surgery (t1), and 1 (t2) and 2 (t3) months after surgery, respectively. Three regions of interest (ROIs) from mesial, distal, and apical sites of the implants were chosen and fractal analysis (FA) was conducted with the box-counting algorithm using White and Rudolph’s method.

**Results:**

A total of 39 patients 19 women and 20 men, with a mean age of 52.2 years (52.3 and 52.1 years, respectively) were included. The mean, minimum and maximum values of mesial (roi1), distal (roi2), and apical (roi3) surfaces were compared. The fractal dimension (FD) values of t1 were significantly lower compared with t0 as they decreased during the first week. FD values gradually increased after the first week although never exceeded the FD values of t0. Also, difference between mean FD values of t0 and t3 were found statistically significant (*p* < 0.05).

**Discussion:**

FA is a promising and noninvasive method to predict osseointegration of a dental implant based on dental radiographs, and it can help shorten the total treatment time.

## Introduction

Dental implants have been used to restore total or partial edentulous jaws for years. Success of dental implants is related to non-complicated surgery and uneventful osseointegration period which depends on patient-related parameters, including general health, implant type, and the quality and quantity of the relevant bone tissue [1]. The quality of bone tissue is closely related to the implant success, there are clinical studies that showed a higher survival rate in the mandible when compared to the maxilla [2–4]. Osseointegration protocols indicate that the implants should receive no loading during this period, generally 3 to 4 months in the mandible and 6 to 8 months in the maxilla [5–7].

One key factor for a successful surgery is the mechanical performance of the bone while the key factor of prosthodontic procedure is effective osseointegration [8]. Also, primary stability and successful osseointegration period depend on factors such as surgical procedure, implant surface, and characteristics. The implant industry and research groups have been trying to shorten the osseointegration period with modification of threads (aggressive or passive implants) and surface coatings or surface roughening with different techniques like titanium plasma spray (TPS), hydroxyapatite coating, or storing the implant in a liquid to reduce the titanium surface’s contact with oxygen.

Some diagnostic tools have been designed for evaluating bone quality, osseointegration, and primary stability of a dental implant [8]. The dual-energy X-ray absorptiometry (DEXA) is the gold standard for the evaluation of bone mineralization. Also, panoramic radiography and Cone-beam computed tomography (CBCT) are advanced imaging modalities that have clinical applications in the field of implant surgery [9]. DEXA cannot provide cross-sectional image and is not as common as CBCT. Therefore, CBCT is more commonly used for evaluating bone mineralization [9]. In the literature, mandibular cortical index (MCI), resonance frequency analyses (RFA), implant stability quotient (ISQ), and fractal dimension (FD) have been described to evaluate bone quality and primary stability before surgical intervention [1, 7].

Fractal analysis is used to describe and measure the morphology of the natural world. For example, FA has been applied to describe dripping taps, stock exchange prices, cell outlines, pulmonary branching, heartbeats, and temporomandibular joint sounds [10, 11]. Mandelbrot first introduced the concept of fractal analysis (FA) [12]. In addition, as some researchers have already suggested that fractal analysis of alveolar trabecular bone could be used as a diagnostic tool to characterize alveolar bone, objectively [13].

Cancellous alveolar bone is composed of interconnected trabecular structures with an underlying geometric pattern which is useful for defining a fractal pattern. FA is a mathematical method that describes complex shapes and structural patterns [14]. FA determines the complexity of the structures quantitatively. The FA of radiographs has also been found to reflect the partial demineralization of the bone tissue [15]. Periapical and panoramic radiographs (OPG) can be used to evaluate the local bone pattern with FA. The aim of this study was to evaluate the effectiveness of FA on determining osseointegration of dental implants.

The null hypothesis of this retrospective study was that if the calculating of bone mineralization on pre-operative and post-operative 2nd month OPG shows similar results, then the FA could indicate the successful osseointegration by around the dental implants.

## Materials and methods

### Patients

This retrospective study was approved by the Ethics Boards and Commissions of Erciyes University, Kayseri, Turkey (2019/456). Out of the radiographic records of patients who had implant surgery only the mandibular premolar/molar implant sites were included in the present study in order to eliminate the superimposition of anatomical entities such as maxillary sinus and hard palate. The inclusion criteria follow as having both pre- and post-implant surgery panoramic radiographs available. These radiographies should be taken before implant surgery (t0), within a week of surgery (t1), and 1 (t2) and 2 (t3) months after surgery, respectively.

Patients were excluded from the study if they had; dental implants which were placed in the anterior region, implant placement with bone graft, implant placement with sinus lift, non-submerged implants, immediate implant placement after extraction, and immediate loading of implants. In addition, patients who had a systemic disease or patient using medication that affected bone metabolism were excluded. Implants were placed by the same surgeon within the recommendation of the dental implant manufacturer. Healing caps of the submerged implants were placed 3 months after waiting for osseointegration period and patients were referred to the Department of Prosthodontics for definitive treatment.

### Radiological analysis

OPGs were taken with the same OPG device (OP200 D; Instrumentarium Dental, Tuusula, Finland; 66–85 kVp, 10–16 mA, 14.1-s exposure time) and the same protocol. Subjects were placed following the manufacturer’s recommendations, the Frankfurt horizontal plane was parallel to the horizontal plane, and the sagittal plane was aligned with the vertical line which was produced by the device. The images were then exported as TIFF files with a resolution of 5.5 LP/mm. The size of the images was 2976 × 1536 pixels.

Three regions of interest (ROIs) from mesial, distal, and apical sites of the implants were chosen on four consecutive OPGs according to a study by Zeytinoğlu et al. ROIs were chosen carefully with the polygon tool of the software to consist of the maximum available area close to the implants without including the roots, lamina dura, or periodontal ligament [16]. Pre-implant and post-implant OPGs were compared and the ROIs in the pre-implant OPGs were placed in similar regions.

**Fig. 1:**
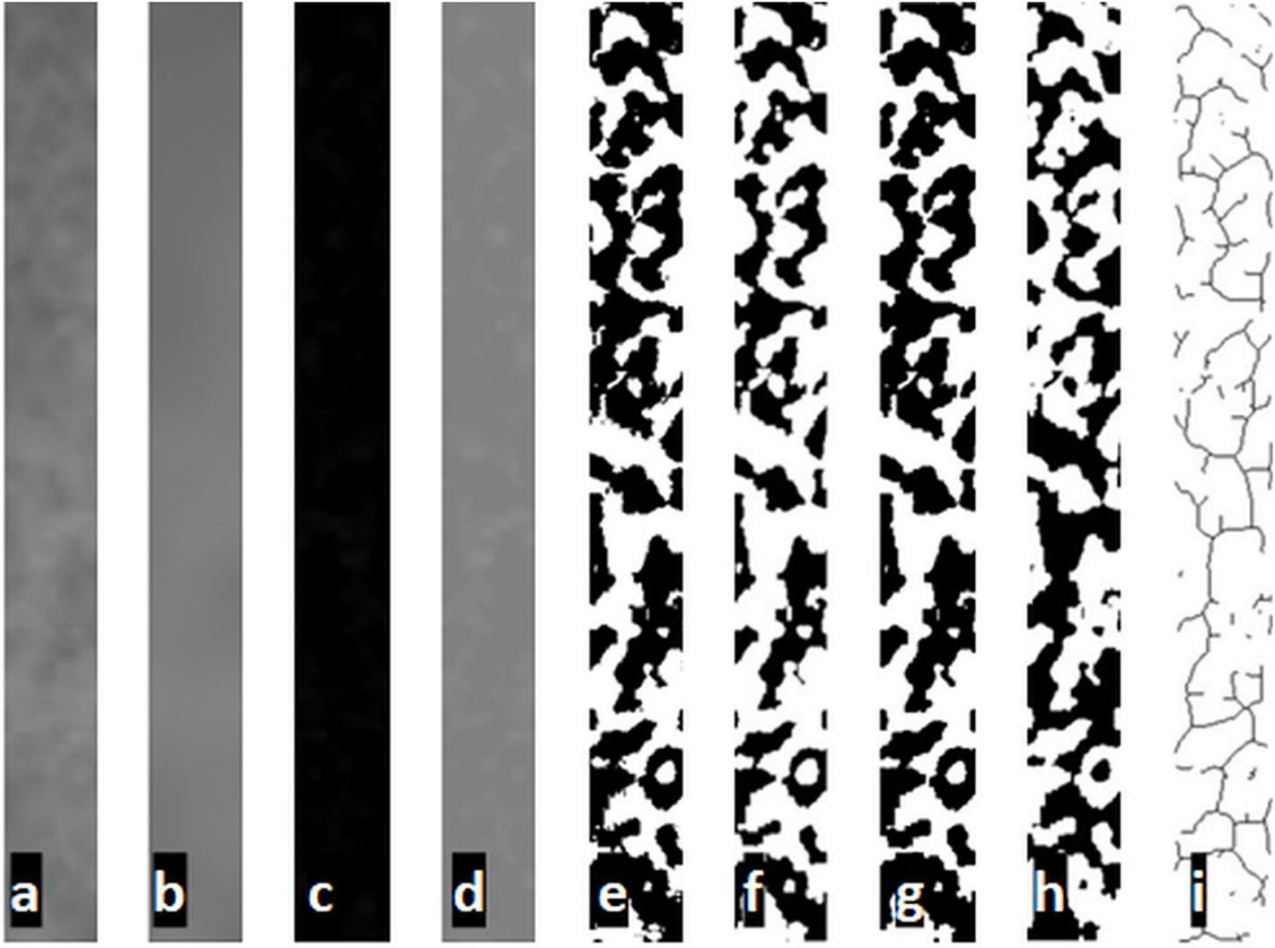
**a** Cropped and dublicated ROI. **b** Blurred version. **c** Subtracted version. **d** Addition of 128 grey value. **e** Binarized version. **f** Eroded version. **g** Dilated version. **h** Inverted version. **i** Skeletonization

Fractal analysis was conducted with the box-counting algorithm using White and Rudolph’s method [17]. Firstly, the chosen ROI was cropped and duplicated. The duplicated image was blurred using Gaussian blur, with this step large-scale variations of brightness caused by the thickness of the object or soft tissue were eliminated. The blurred version was subtracted from the original version. A gray value of 128 was added to each pixel location, resulting in a new image with a mean pixel value of 128. Thus, different variations in the image could represent the different types of features with specific brightness (trabeculae and bone marrow). The resultant image was made binary with the threshold tool of the software with a brightness value of 128. Values which were equal or smaller than the 128-pixel value were converted to black while the other values were converted to white. The image was detached into two areas representing the trabeculae and bone marrow. The image was eroded and dilated for reducing the noise. The resulting image was inverted; thus, parts representing the trabecular bone were changed to black, and part representing the bone marrow was changed to white. The final process was skeletonization; the image was eroded until the only central line of the pixels remained. Fractal dimension values were calculated with the box-counting function of the software. Squares of 2-, 3-, 4-, 6-, 8-, 12-, 16-, 32-, and 64-sized pixels were placed on the image. The number of squares that included the trabeculae, and the total count of the squares were measured for each different sized pixel. The logarithmic scale graph of the values was drawn (Fig. 1a, i).

**Fig. 2:**
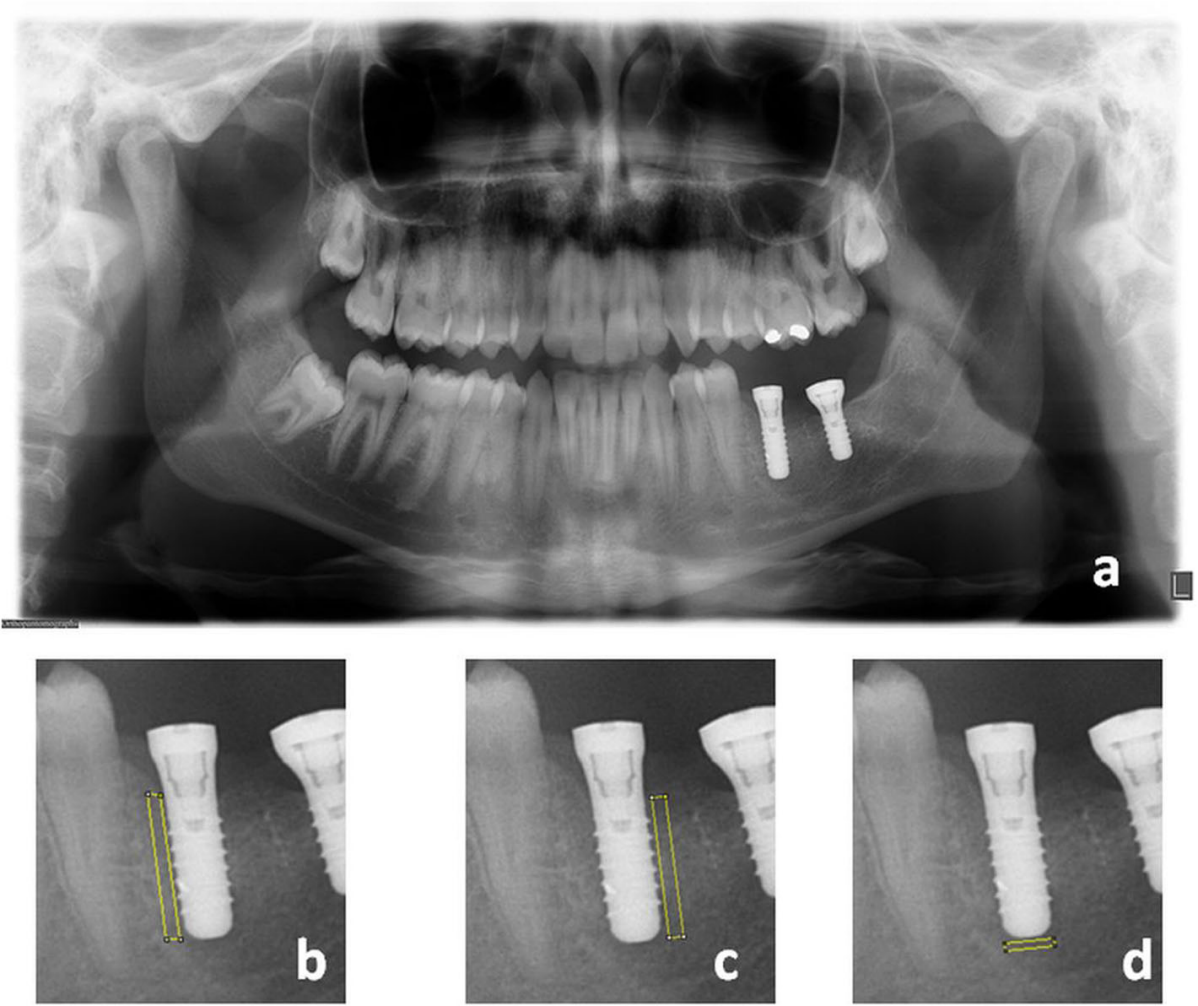
**a** Selection of ROIs. **b** Selection of roi1. **c** Selection of roi2. **d** Selection of roi3

Fractal dimension value was calculated by measuring the slope of the line which was formed aligned to the plotted points on the graph (Fig. 2a–d).

### Statistical analysis

Descriptive analyzes were performed to give information about the general characteristics of the working groups. Data of continuous variables are in the form of mean ± standard deviation; data on categorical variables are given as *n* (%). When comparing the averages of repetitive quantitative variables between groups, a two-way analysis of variance is used in repeated measurements. Bonferroni correction is used for multiple comparisons. When *p* values were calculated less than 0.05, it is considered statistically significant. A software package was used (IBM SPSS Statistics 19, SPSS inc., An IBM Co., Somers, NY) for statistical analysis.

## Results

Thirty-nine patients, 19 women and 20 men, with a mean age of 52.2 years (52.3 and 52.1 years, respectively), met the inclusion criteria. Sixty-six implants placed in the premolar and molar regions of the mandible were evaluated in this retrospective study. All implants had a cylindrical body with similar dimensions: diameters of 3.7–4.1 mm and implant lengths of 8–11.5 mm. Early implant failure did not occur with parafunction, infection, or immediate loading, and all implants were osseointegrated at the end of 3 months. The arch configuration of all patients was mandibular Kennedy Class I.

**Fig. 3:**
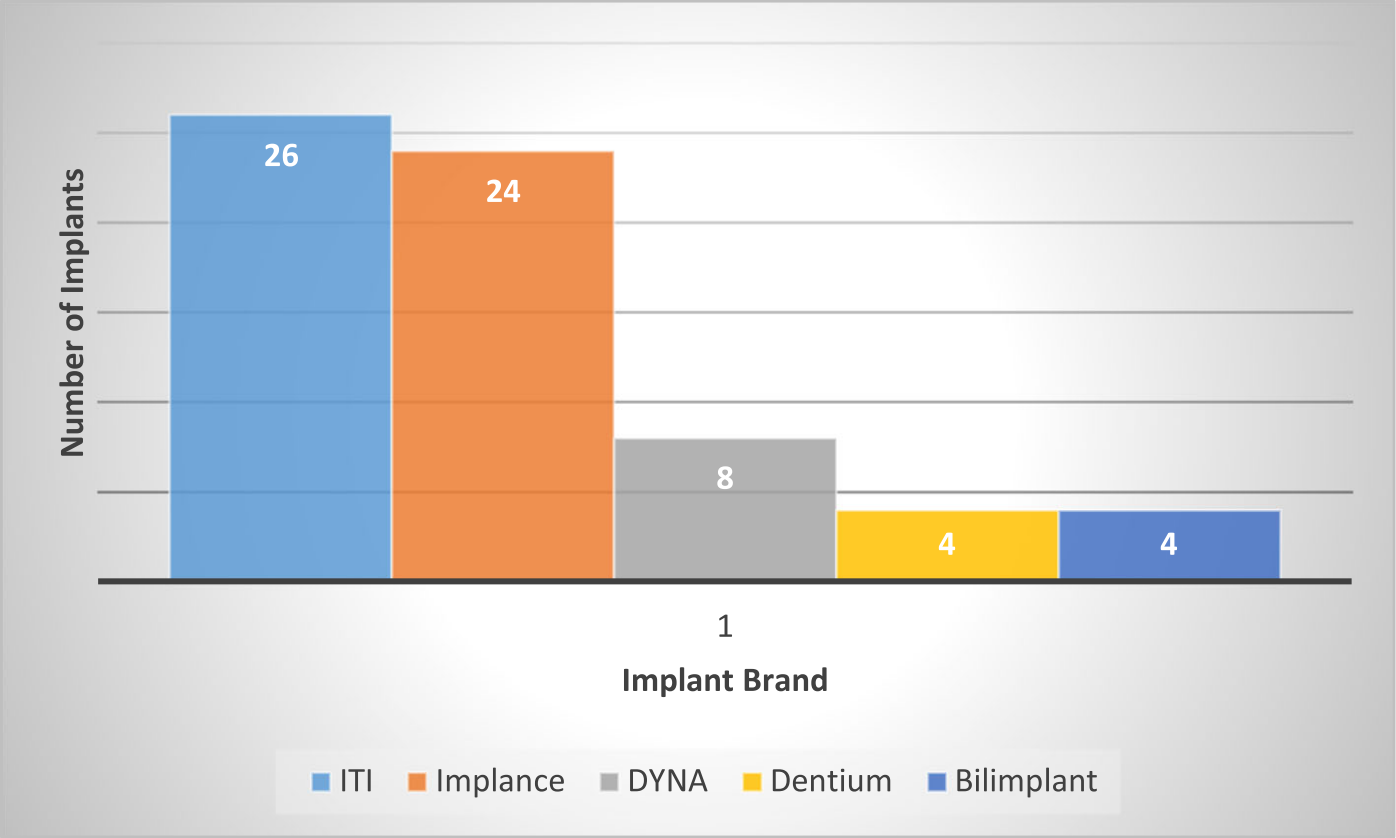
Numbers and Manufacturers of included implants

The brands of the dental implants included in the study is shown in Fig. 3.

**Fig. 4:**
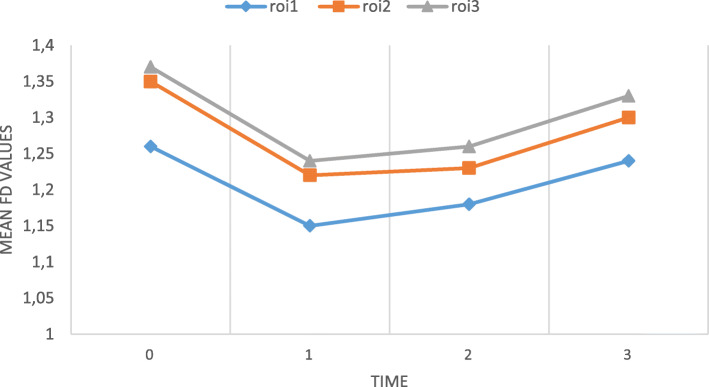
Mean FD values of the implants. (t0: before implant; t1: 0 to 7 days after surgery; t2: 1 month after surgery; t3: 2 months after surgery, roi1: mesial surface; roi2: distal surface; roi3: apical surface)

FD values at mesial, distal, and apical regions of implants were calculated on panoramic X-rays (Fig. 2). The mean, minimum, and maximum values of mesial (roi1), distal (roi2), and apical (roi3) surfaces were compared in Fig. 4. The FD values decreased immediately after the operation (t1 and t2 roi) and increased gradually according to the time lapse.

Gender based comparison of FD values is shown in Table 1. No significant differences were found in terms of gender. In the roi1 variables, there was a significant relationship between FD values at t0 and t2 (*p* < 0.05). Also, FD values of roi2 and roi3 were similar. There were no significant differences between t1 and t2 FD values. The mean FD values for all surfaces at t0, t1, t2, and t3 were 1.33, 1.2, 1.22, and 1.29, respectively. Difference between mean FD values of t0 and t3 were statistically significant (*p* < 0.05).

**Table 1:** Values presented with mean and standard deviation

Variables	Total	Sex		p_1_
		Female	Male	
t0 roi1	1.26 ± 0.11 (a)	1.27 ± 0.11 (a)	1.25 ± 0.11 (a)	0.403
t1 roi1	1.15 ± 0.11 (b)	1.14 ± 0.09 (b)	1.16 ± 0.13 (b)	0.603
t2 roi1	1.18 ± 0.11 (b)	1.17 ± 0.09 (b)	1.19 ± 0.13 (ab)	0.371
t3 roi1	1.24 ± 0.11 (c)	1.25 ± 0.1 (a)	1.23 ± 0.11 (a)	0.522
*p*_*2*_	< 0.05	< 0.05	< 0.05	
t0 roi2	1.35 ± 0.1 (a)	1.35 ± 0.09 (a)	1,35 ± 0.11 (a)	0.960
t1 roi2	1.22 ± 0.14 (b)	1.2 ± 0.11 (b)	1,24 ± 0.17 (b)	0.306
t2 roi2	1.23 ± 0.13 (b)	1.22 ± 0.11 (b)	1.25 ± 0.15 (b)	0.341
t3 roi2	1.3 ± 0.1 (c)	1.31 ± 0.07 (c)	1.29 ± 0.12 (b)	0.489
*p*_*2*_	< 0.05	< 0.05	< 0.05	
t0 roi3	1.37 ± 0.08 (a)	1.37 ± 0.09 (a)	1.37 ± 0.07 (a)	0.911
t1 roi3	1.24 ± 0.11 (b)	1.23 ± 0.12 (b)	1.25 ± 0.1 (b)	0.435
t2 roi3	1.26 ± 0.11 (b)	1.24 ± 0.1 (b)	1.29 ± 0.1 (bc)	0.071
t3 roi3	1.33 ± 0.09 (c)	1.34 ± 0.1 (c)	1.32 ± 0.07 (c)	0.622
*p*_*2*_	< 0.05	< 0.05	< 0.05	
t0 FD_mean_	1.33 ± 0.07 (a)	1.33 ± 0.07 (a)	1.32 ± 0.08 (a)	0.629
t1 FD_mean_	1.2 ± 0.09 (b)	1.19 ± 0.07 (b)	1.22 ± 0.1 (b)	0.264
t2 FD_mean_	1.22 ± 0.08 (b)	1.21 ± 0.06 (b)	1.24 ± 0.1 (b)	0.094
t3 FD_mean_	1.29 ± 0.08 (c)	1.3 ± 0.07 (c)	1.28 ± 0.08 (c)	0.431
*p*_*2*_	< 0.05	< 0.05	< 0.05	

Repeated measures ANOVA analysis was used. *t0* before implant, *t1* 0 to 7 days after surgery, *t2* 1 month after surgery, *t3* 2 months after surgery, *roi1* mesial surface, *roi2* distal surface, *roi3* apical surface, *p*_*1*_ comparison between groups, *p*_*2*_ in-group comparison, *(abc)* different letters in the same column represent statistical significance

## Discussion

The osseointegration concept is defined as the successful and functional direct connection between bone and implant surface by Brånemark [18]. The osseointegration process depends on factors such as biocompatibility of the material, macrostructure, and microstructure of the implant, surgical technique, bone quality, and loading. At the time of placement, primary stability of a dental implant has been considered a prerequisite for its survival [19].

In the literature, the primary stability has been evaluated by using RFA, ISQ, MCI, and FA. RFA and insertion torque (IT) are commonly use to evaluate the primary stability. RFA was firstly used in dentistry by Meredith in 1996 to measure the primary stability [20]. RFA allows to control implant stability non-invasively throughout the entire healing period, although it is not standardized on different implants. The ISQ allows to measure implant stability and bone quality, and provides information about the implant’s loading time. MCI is a well-known diagnostic tool for the assessment of osteoporotic patients [21]. Also, MCI evaluation before implant surgery may provide useful information about the bone quality. There are very limited number of studies that explain the association between ISQ, MCI, and FA. Tözüm et al. concluded that MCI may provide a treatment plan before surgery and FA may be a useful method for understanding the healing process around implants and implant stability [22].

FA was described by Sanchez and Uzcategui to evaluate and analyze the bone pattern and implant structure in dentistry [23]. In the medical field, FA is currently employed for evaluating patterns or texture which is the ability to model complex structures. Also, FD have been used to identify abnormal vascular patterns, tumor-associated neovascular growth, pulmonary branching, heartbeats, dripping taps, stock exchange prices, and temporomandibular joint sounds [11, 12]. Also, FA is used to determine the changes in alveolar bone structure following immediate implant placement and immediate temporary restoration. Lee et al. found a positive relationship between the ISQ measured after implant placement and the FD value measured prior to placement [1]. Considering these studies suggest that FA might be a useful tool to determine the primary stability of dental implants and the quality of the local bone surrounding the implant.

Koh et al. showed that the evaluation of FD from panoramic radiographs is most reliable in the mandibular premolar region, as trabecular pattern could be seen clearly in dense bone [24]. Therefore, in present study, the implants placed in mandibular premolar and molar regions were included. Also, OPGs were used to determine the FD, because they were routinely taken as an institutional protocol. There are many studies in the literature that evaluate bone quality, peri-implantitis, primary stability of the implant and resorptive changes in the bone as a result of early loading using MCI, RFA, FD, ISQ. However, very few studies have attempted to establish a correlation between before and after the operation. The present study investigated whether the FD value of the bone before the implant placement correlated with the FD values of the bone 1 week, 1 month, and 2 months after implant placement.

Sansare et al. showed that the FD value was significantly increased after implant placement, due to increase amount of bony microstructure and bony trabeculae around the implant [25]. In present study, the FD values of t1 was significantly lower compared with t0 as they decreased during the first week. FD values gradually increased after the first week although never exceeded the FD values of t0. Although, there was a statistically significant difference between t0 and t3 values, the mean FD values of t3 were quite close to the FD values of t0. The results of present study showed that FD could be used as a predictor for defining the osseointegration via OPGs. Similarly, Heo et al. reported that FD decreased in the first 2 days after the ortognatic surgery and FD increased gradually according to the time lapse [26]. This can be explained by the healing pattern of the bone. Ellis et al. reported that after ortognatic surgery the osteotomy line could be healed by bone formation which was filled with mature bone 6 weeks post-surgery [27]. Thus, it could be assumed that the FD reflected an increase on the bone formation and the trabecular pattern after bone healing process. In present study, significantly increased fractal dimensions were observed after a 2-month healing period.

The implant loading protocol suggests loading at least 8 weeks after implant placement to reduce all complication and risks [28]. Bornstein et al. showed a successful bone tissue integration with the early loading protocol that is observed after 3 weeks of healing period by monitoring the primary stability with ISQ [29]. Balshi et al. concluded that an immediate loading protocol should have a period of healing for the first 2 months after implant placement. Also, they found that the RFA showed a decrease in bone-implant stability within first month of surgery despite increased stability within second and third months [30]. In present study, fractal dimensions show that the osseointegration and bone healing process can be completed in 2 months.

The osseointegration of dental implants depend on patient-related factors such as bone metabolism [31] and bone metabolism differs between male and female genders in terms of bone regulating hormones. In previous studies, the success of osseointegration was correlated with the density and quality of bone in osteoporotic patients [32, 33]. Also, orthopedic diseases are prevalent in women such as osteoporosis and osteoarthritis. August et al. showed that the estrogen deficiency and the resultant bony changes may be risk factors for endosseous implant failure [34]. Chen et al. showed that the implants in women had lower ISQs compared with men; however, the difference was not statistically significant [35]. There are no other studies in the literature that compare the preoperative and postoperative implant stability with FA by gender. In present study, there was no statistically significant difference between genders (19 women and 20 men, with a mean age of 52.3 and 52.1 years, respectively).

There are some limitations of this study; due to the retrospective design of the study total numbers of the included implants were low and no additional measurements were performed. However, considering that only the implants with a survival of at least 1 year were included in the study, it could be said that all implants were osseointegrated at the third month which is also the time of the placement of healing caps. Additionally, as different implants with different surface characteristics were included in this study, FA values of the different surfaces were not compared. Hence, as confirming the hypothesis of present study, the biggest takeaway of this study was the consideration that the FA could predict osseointegration of a dental implant.

As a conclusion, FA is a promising, reliable, and noninvasive method to predict osseointegration of dental implants based on two-dimensional dental radiographs, and it can help to shorten the total treatment time. However, future prospective studies with large number of implants that investigate the relationship between FA and osseointegration period and ISQ values (at placement, at second, and third months) are needed.

## Data Availability

The datasets used and/or analyzed during the current study are available from the corresponding author on reasonable request.
